# Pyorrhœa Alveolaris

**Published:** 1899-07

**Authors:** Frederick W. Allen

**Affiliations:** University of Pennsylvania


### PYORRHŒA ALVEOLARIS.[1]

BY FREDERICK W. ALLEN, UNIVERSITY OF PENNSYLVANIA.

CASE.—Miss Mary Q., aged forty-one, presented herself at clinic for treatment. Patient was of a nervo-bilious temperament.

*Diagnosis.*—It was evident that the patient was affected by pyorrhœa. The teeth were generally involved, pus being noticeable

---

[1] This and the following cases of treatment of pyorrhœa alveolaris are published with the view of answering the question, so frequently made, "Can pyorrhœa be cured?" Each member of the senior class of the Dental Department of the University of Pennsylvania was required to treat a pathological case, and was given full liberty to use his own judgment in the therapeutical management. The cases were all registered and examined from time to time by the teachers in charge.—ED.

at the gingival borders, and upon pressure there was a considerable discharge. The gums were tumid, and the lower anterior centrals and laterals were denuded to half the length of the roots, the teeth being quite loose. The upper centrals, while not denuded of their gingivæ, had pus-pockets extending nearly to their apices. There were pockets on nearly all the teeth, and generally containing darkened serumal calculi, firmly adherent to the roots. The mouth was in a very unhygienic condition, and the offensive odor peculiar to such cases was especially noticeable.

*Prognosis.*—Was favorable, depending upon the successful removal of the exciting and predisposing causes.

*Treatment.*—As the mouth was in a very unclean condition, there being considerable deposits of salivary calculi upon the teeth, the tongue heavily coated, the breath was made additionally offensive by a gastric condition. The patient was given a mouth-wash of permanganate of potassium and water. This was used until the mucous membrane of the mouth took on a dark-brown color. Then a wash of hydrogen dioxide was given, diluted with three times its bulk in water, to which was added sufficient soda bicarbonate to neutralize the preserving acid of the dioxide. These washes softened the deposits upon the teeth, corrected the fetor of the breath, and removed the *débris* from the tongue and mucous membrane. In this way the scaling was performed with less injury to the tissues, and the field of operation was left more desirable for treatment. The teeth were then carefully scaled and polished, and in removing the serumal deposits from the roots of the teeth thin, flexible, Cushing's scalers were used, with the "pushing motion." The pockets and necks of the teeth were syringed with warm water. Then a three-per-cent. solution of hydrogen dioxide was introduced, removing the pus, foreign bodies, micro-organisms, etc. The *débris* was washed out with tepid water. Then a twenty-five-per-cent. solution of sulphuric acid (commercial) was applied to the necks of the teeth and introduced into the pockets by means of Japanese tooth-picks, care being taken to avoid applying the acid to the crowns of the teeth and not to use a surplus. The acid was allowed to remain for about one minute, carbonizing the tissues. It was then neutralized with soda bicarbonate, which produced slight ebullition; the parts were then thoroughly washed out with warm water, and were practically free from germs and all foreign bodies. The pockets were then packed with sulphate of quinine, which not only closed them, acting to prevent reinfection upon the lower surfaces, but being locally a protoplasmic poison, preventing the emigration of the leucocytes, and being also an antiseptic.

The patient was then dismissed for two days, and given a prescription of

℞ Hydronaphthol, gr. xv;

Alcoholis,

Aquæ destillatæ, āā ℥i. M.

Sig.—Twenty drops in a tumbler of water. Use twice a day as a mouth-wash.

Upon return the pockets were washed out with hydrogen dioxide and additional quinine packed in, care being taken not to disturb the tissues. After seven days additional treatment was made, and at the end of three weeks new tissue had nearly filled the former pus-pockets, with the exception of the centrals, which were again treated with the sulphuric acid.

Continuing the same treatment of the hydrogen dioxide and quinine, granulations commenced in the lower centrals, and after three months' treatment the teeth were quite firm in their sockets, and in five months new tissue had formed around the necks of the teeth.

The patient was then dismissed, with instructions for the proper care of the mouth, and to continue with the use of the mouth-wash.

Coincident with the removing of the pyorrhœal condition, the patient was relieved of a former gastric condition.

NOTES.—Having previously treated three cases, the patients being older, ranging from fifty to sixty-five years of age, the results were not so favorable. One was treated in connection with a local treatment, together with a large quantity of water, taken daily, together with bitartrate of lithia. This case apparently yielded, although, upon cessation of treatment for six weeks, it returned to the original condition.

In another case the protoiodide of mercury was used, but the condition of the alveolus was such that the teeth could not regain firmness in the jaw. The other case, sixty-five years of age, was, upon further investigation, found to be a case of senile dentine, and a device was made to retain the teeth to a proper time to make an artificial denture.

